# Analytical Framework to Navigate Microalgae-Based Product Development—Aligning Commercialization and Regulatory Pathways

**DOI:** 10.3390/md24020066

**Published:** 2026-02-03

**Authors:** Galey Tenzin, Kira Schipper, Harshit Rathore, Hemil Shah, Edgar Brea, Ben Hankamer, Damian Hine

**Affiliations:** 1 Queensland Alliance for Agriculture and Food Innovation, The University of Queensland, Brisbane 4072, Australia; 2 Institute for Molecular Bioscience, The University of Queensland, Brisbane 4072, Australia; 3 UQ Business School, The University of Queensland, Brisbane 4072, Australia

**Keywords:** microalgae innovation, speed-to-market, competitive edge, new product development, market economics, navigating regulatory pathways, multicriteria decision-making

## Abstract

After numerous false starts, the global microalgae industry is re-emerging, driven by its potential to address critical challenges in food and nutrition, sustainable energy, nutraceuticals, cosmetics and pharmaceuticals, and climate change mitigation. Although technical advances in microalgae production show value adding potential, progressing from innovation to product launch and competitiveness is complex. It requires an integrated understanding of technology readiness, regulatory compliance, financial necessities, and market competition. This study presents a novel analytical framework underpinning a data-enabled, evidence-based approach to navigating the innovation pathways to market and beyond. The framework integrates value-add opportunities, identifying key stages faced in pre-competitive (including Technology Readiness Level (TRL), R&D spend, and patent trends), and competitive market stages (including product launches, product claims, market size, market share, growth/maturity, international markets, distribution channels, sectoral profile, and competitive landscape), aligned with regulatory requirements. Although not without limitations, such as incomplete data for emerging products, as well as reliance on secondary sources for product stage determination and market size estimates which can influence the accuracy of TRL classification and market potential estimates. This integration of multiple analyses can help in identifying market opportunities and business competitiveness via product, business, and industry level analyses in the pre-competitive (pre-market launch) and competitive (on market) landscapes. Building on the team’s interdisciplinary experience of developing interactive dashboards for food and beverage industries, and microalga processes, this paper provides an overview of the framework, which was designed to guide businesses and researchers in an emerging microalgae industry through the complex landscape of product development along regulatory and commercial pathways.

## 1. Introduction

Microalgae comprise a highly diverse group of photosynthetic microorganisms distributed across marine, freshwater, and terrestrial environments. Although biologically distinct, the algae industry conventionally groups both microalgae (eukaryotes) and cyanobacteria (prokaryotes) under the broader term microalgae, including widely cultivated genera such as *Limnospira* (more commonly referred to as Spirulina). In this paper this broader industry definition has been adopted). The recorded diversity in microalgae continues to grow, with more than 180,000 species recorded to date [1]. Microalga’s commercial versality stems from their biochemical capacity to produce proteins, pigments, lipids, polysaccharides, fatty acids, and nanomaterials using only sunlight, CO_2_, and basic macro- and micronutrients [2]. Owing to this metabolic flexibility, microalgae are increasingly recognized as important contributors to the emerging global bioeconomy, with applications spanning food, feed, biofuels, health, and nutraceutical products in addition to environmental applications such as wastewater treatment and carbon sequestration.

Commercial interest dates back to the mid-20th century, as a “new source of food”, highlighted by the 1950 “Algal Mass Culture Symposium” at Stanford University [3,4]. This was followed by an increased interest as a source of biofuel in the 1970s and more substantially in the 2000s, driven by energy security concerns and climate change mitigation goals.

Today, commercial production is dominated by a small number of well-established species—*Limnospira* sp., *Chlorella vulgaris*, *Dunaliella salina* and *Haematococcus pluvialis*—which have proven amenable to large scale cultivation and established regulatory pathways [5]. Despite significant progress in the mid to high value product range from these species (e.g., nutraceuticals, health foods), impactful lower value products, such as renewable fuels, have struggled to meet market expectations with the available technology [4]. The consequent downturn has catalyzed a strategic pivot toward diversified applications beyond fuels, including high-value products (e.g., recombinant proteins, cosmetics, nutraceuticals) and mid value products (e.g., food, animal feed, biopolymers, and nanomaterials), opening opportunities to expand current production and develop new markets [6]. Today, microalgae innovation increasingly emphasizes integrated biorefineries and multi-product value chains, aiming to maximize economic viability while supporting sustainability objectives [6].

As global priorities shift towards decarbonization and sustainable, value-added production, light driven microalgae-based biomanufacturing is emerging at the intersection of biotechnology, environmental protection, industrial innovation, and sovereign security (e.g., food and fuel security) [7]. While these applications are gaining traction, the transition from promising concepts to competitive large-scale production remains challenging. Despite substantial technical advances, scaling bio-industrial manufacturing processes from lab to industrial scale is fraught with risks and uncertainties [8]. Many microalgae-based bioenergy systems, for example, remain at mid-range Technology Readiness Levels (TRLs), limiting their rapid or widespread implementation [9,10]. This reflects persistent technical and economic barriers, such as complex cultivation processes, limited pilot-scale data (e.g., on fully automated processes), and insufficient policy support, which hinder scale-up prospects and delay commercialization [11]. Compounding these challenges, many ventures delay integrated market analysis and regulatory planning until late development, reducing their ability to capture value and achieve competitive positioning. As a result, ventures often struggle to transition beyond pilot/demonstration scale stages due to limited understanding of how to match their innovation to market needs and deliver products optimized for the market, and capable of entering and growing in niche markets, whilst complying with regulatory requirements.

Scaling production and achieving market competitiveness requires robust economic and regulatory frameworks that can sustain innovation and unlock new opportunities [12]. However, these frameworks alone are not sufficient. Overcoming existing barriers also demands early alignment of technical, market, and regulatory strategies. To support this, the microalgae industry needs tools that not only enable anticipation of competitive threats and formation of collaborative partnerships, but also facilitate the development of compliant, high quality, sustainable, and cost-effective manufacturing practices and supply chains, while adapting to disruptive technologies and evolving regulatory requirements. The objective of this work is therefore to introduce a unified analytical framework that integrates biological, technical, regulatory, and commercial considerations into a single structured approach. This framework is proposed to guide and assist businesses and researchers in navigating complex innovation pathways, ensuring that product development aligns with market readiness and regulatory requirements from the earliest stages.

## 2. Framework Development

Underpinning the developed framework, this paper presents an integrated dashboard to serve as a tool for scientists, researchers, companies, and industry stakeholders to systematically assess microalgae innovations and the current and near future environments they are facing. It starts with value-add opportunities for products and technologies in development which have not yet reached the market. The dashboard integrates and extends the underlying conceptual frameworks of Technology Readiness Levels (TRLs), new product and process development, business model innovation, and market economics included in the principles of the Gartner Hype Cycle.

The Hype Cycle is a conceptual framework introduced by Gartner Inc. in 1995 that serves as a useful model to define and forecast technological development and adoption [13]. It maps the trajectory of technological innovation from inception to mainstream adoption by tracing innovations as they pass through different stages of technical capability, investment horizons, market adoption, and public expectations over time [14]. It highlights the typical trajectory of a technology in terms of expectations and the viability of its value over time [15]. Early-stage innovations often experience an “innovation trigger”, that generates initial excitement and inflated expectations followed by disillusionment, which marks a critical inflection point where initial enthusiasm wanes, and practical limitations surface, especially the loss of financial support due to failure to meet development milestones that would trigger further investment, before reaching a plateau of productivity, when technologies achieve maturity with widespread adoption and regulatory norms stabilize [14,16]. The Hype Cycle can help signal investor resilience while TRLs indicate the readiness of technology for scaling.

Integrating the Hype Cycle with TRLs provides a practical foundation to navigate the commercial and regulatory complexities of new product development. The two frameworks allow firms to view adoption trajectories more accurately, avoid premature or late market entry, and align product development with realistic timelines. They allow firms to better anticipate regulatory hurdles and market dynamics, fostering resilient innovation pathways. Technological readiness and maturity can be assessed through Technology Readiness Levels (TRLs). The TRL scale spans the continuum from fundamental research to full-scale implementation, which ranges from initial idea (TRL 1) to full commercial deployment (TRL 9). Developed by the National Aeronautics and Space Administration (NASA) to manage technological risks in space missions, the TRL scale provides a standardized framework to systematically assess the maturity of emerging technologies [17]. The metric assists policymakers, investors, and innovators with identifying a technology’s stage of development, thereby offering a practical tool for navigating time to market and hype cycles. The framework presented in this paper enables a phased evaluation of technical feasibility, scalability, and market potential. This alignment supports strategic planning for scale-up and commercialization.

Regulatory compliance is also a critical determinant of market access. To ensure that innovations are not only technically viable but also legally marketable [18], the framework also embeds a parallel regulatory pathway that identifies key regulatory requirements and compliance checkpoints along the development pipeline. Included are regulatory pathways that impact research, as well as large-scale cultivation, processing, and trade of microalgae and their derivatives in Australia. It seeks to pinpoint the relevant legislation and regulations to help industries navigate regulatory complexity and identify pathways to compliance.

**Figure 1:**
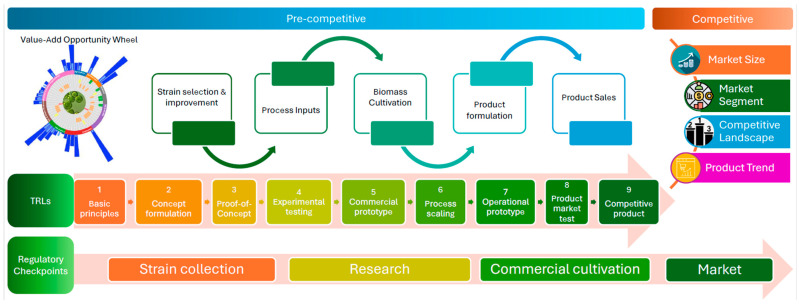
Conceptual framework for an interactive dashboard supporting microalgae product development. The dashboard integrates pre-competitive and competitive market analytics with regulatory pathways to guide businesses, scientists, and researchers through complex innovation stages. It enables assessment of technology readiness levels (TRLs), market and technology trends, and competitive landscapes, while informing strategies for market entry and product positioning. A more detailed explanation of the Value-Add Opportunity Wheel is provided in Section 3.3.

Figure 1 presents a conceptual framework for the dashboard. By navigating through the relevant dashboard sections, stakeholders can evaluate TRLs, monitor market and technology trends, and the competitive landscape that they are likely to encounter upon market entry. This tool also informs the design of market entry strategies for novel or improved products and assists identification of alternative markets for existing products.

The framework is structured around two interconnected domains: pre-competitive and competitive market stages. The pre-competitive domain encompasses early-stage R&D activities such as strain selection, laboratory and pilot-scale cultivation, harvesting, extraction, and formulation—critical steps for establishing technical feasibility and scalability of microalgae-derived products. The competitive domain focuses on market-facing considerations, including products, regulatory approval, commercial viability, and competitive intensity. By aligning these domains with TRLs 1–9, the framework enables systematic assessment of technological maturity across the development continuum, supporting benchmarking and strategic planning for scale-up and commercialization. Embedded within the structure is a parallel regulatory pathway that identifies critical compliance checkpoints at key junctures—research, large-scale production, and product development and sales—tailored to targeted industries or markets. This integration responds to the reality that regulatory hurdles remain among the most significant barriers to industrial adoption [19].

The team, comprising experts in microalgae, law, innovation and business economics, technology strategy, and data science, conducted an in-depth analysis of production, commercialization, and regulatory aspects of the microalgae industry. Specifically, systematic reviews of scientific publications, patents, product launches, market size, and regulatory frameworks were performed using triangulated data sources to map microalgal technological development and applications across both pre-competitive and competitive phases. These analyses provide signals of changing investment, scientific progress or choke points and competitive dynamics, offering insights into industry trends. Large Language Models (LLMs) were applied to patent product launches and product claims data, enabling businesses to more easily assess current and potential microalgae markets. Although significant gaps persist in available microalgae industry data, in Australia as well as globally, as far as possible, the dashboard interface delivers an integrated approach to support business decision making in the microalgal bioproduct sector. A beta-version of the dashboard was released (the dashboard was released for use by microalgae companies in Australia and, therefore, is not openly accessible) in October 2025.

## 3. Framework Application

Building on the conceptual framework introduced earlier, the dashboard operationalizes the framework by integrating technical, market, and regulatory analytics into a single platform, to deliver evidence-based insights across the innovation lifecycle. It addresses the challenges identified in the introduction—slow and limited TRL progression and technological maturity, regulatory complexity, and market uncertainty.

### 3.1. Overall Dashboard

**Figure 2:**
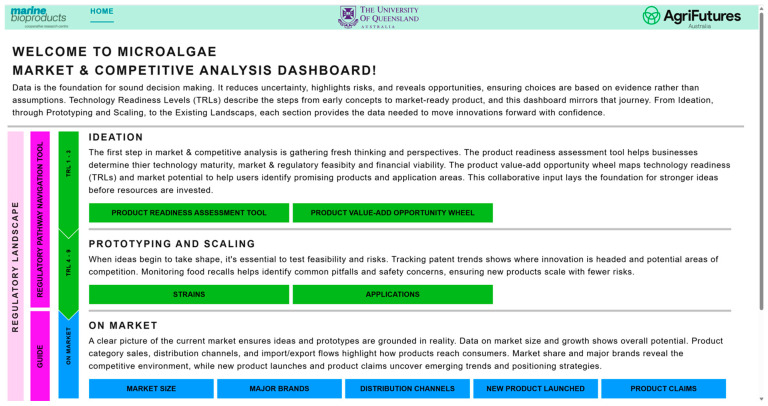
Architecture of the interactive dashboard for microalgae product development, structured around two interconnected domains: pre-competitive and competitive.

Figure 2 illustrates the dashboard’s architecture, structured around two main interconnected domains: pre-competitive and competitive. The pre-competitive domain focusses on early-stage R&D (ideation, TRL 1–3) by visualizing potential microalgae-based (co-products through the Product Value-Add Opportunity Wheel, monitoring patent trends across species and application areas (TRL 4–6), and providing tools to assess product readiness and regulatory compliance (TRL 7–9).

The competitive domain provides market intelligence, including market size, product launch data, claims analysis, and competitive landscape insights with the product readiness assessment tool outlined below. These domains enable users to benchmark their position, explore new product development opportunities, and design regulatory pathways and market entry strategies. This integrated approach ensures that technical progress is aligned with market readiness and regulatory obligations, derisking and potentially accelerating time-to-market.

### 3.2. Product Readiness Assessment Tool

**Figure 3:**
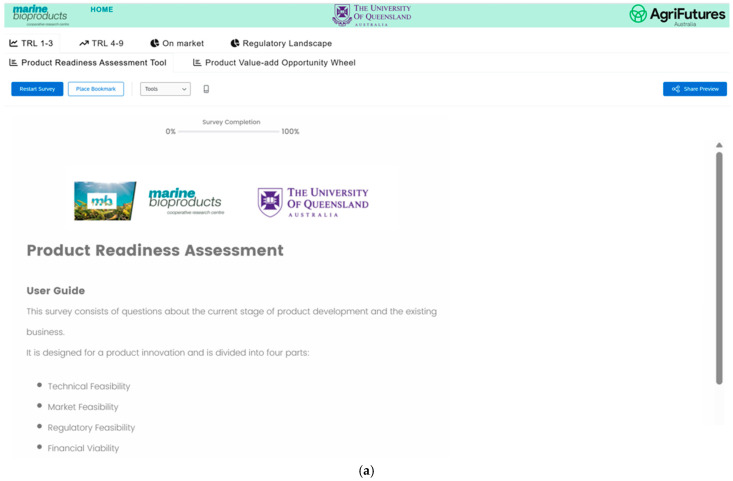

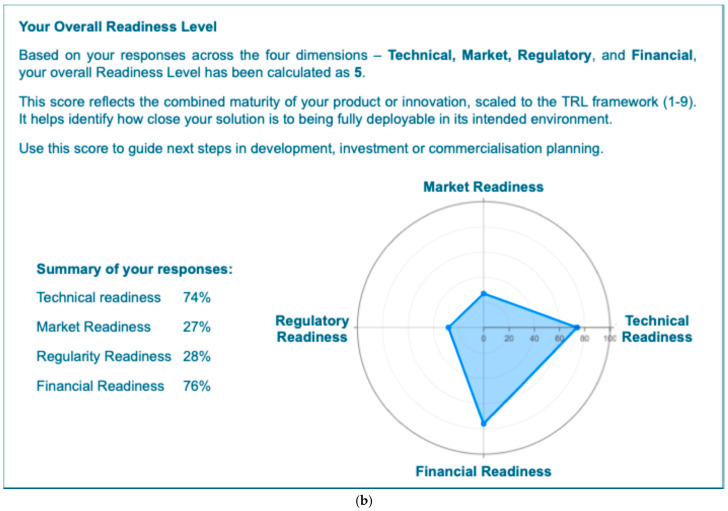
(**a**): Product Readiness Assessment Tool-Landing Page and User Guide (**b**) Product readiness assessment—an exemplar result of the questionnaire showing a start-up scenario, with advanced technical and financial readiness, and gaps in market and regulatory readiness.

The Product Readiness Assessment tool is designed to help businesses evaluate their technology maturity, market and regulatory feasibility and financial viability. The tool (Figure 3a) is an interactive diagnostic questionnaire for businesses to assess their TRL stage. Figure 3b is an exemplar of a product readiness assessment which shows high technical and financial readiness but low regulatory and market readiness. The reality for most businesses is that their weakest domain is their rate limiting step. If all other domains are at high TRLs but one is low TRL, then the business is at low TRL for that specific product, process or technology. This is critical for the speed to market the business wishes to achieve.

### 3.3. Product Value-Add Opportunity Wheel

The Product Value-Add Opportunity Wheel is a core component of the dashboard, designed to map market and product category opportunities across the microalgae innovation landscape. It combines two critical dimensions—technology readiness (TRLs) and market potential—into a single visual representation, enabling users to identify promising products and application areas.

#### 3.3.1. Wheel Development Methodology

To construct the Product Value-Add Opportunity Wheel, a multistep process was applied. First, literature reviews, publications, and online searches through Google (covering publications up to September 2025) were used to identify 61 microalgae derived products with documented R&D activity or demonstrated commercial interest. These were then classified into the most appropriate application area: agriculture, animal feed, biofuels, biomaterials, cosmetics, food and beverages, industrial and chemical products, nutrition and dietary supplements. Environmental applications of microalgae, such as wastewater treatment and carbon capture, although highly relevant and a growing application area, were not included in this aspect as the market value metrics for these services are incomparable with conventional product-based markets and cannot be meaningfully integrated into the value-based comparative framework used for the wheel. Instead, these applications were treated as enabling technologies and ecosystem services rather than discrete market products. Secondly, each product was assigned a development stage based on publicly available evidence (e.g., peer-reviewed studies, company websites, press-releases, demonstration facility announcements) (Table S1 for details), and categorized as: (1) Fundamental Research (TRL 1–2), (2) Applied Research (TRL 3–4), (3) Pilot Stage (TRL 5–7), (4) Precompetitive (TRL 8–9), or (5) Commercial (>TLR 9). Subsequently, product market analyses were conducted. For products already on the market, the algae-based product market size and compound annual growth rate (CAGR) was used. For products still in development, the overall market size and CAGR for the product category were applied as a proxy, drawing on proprietary market research databases and industry reports (e.g., Euromonitor and MarketsandMarkets). Where possible, patent counts for specific algae-based products were added based on single patent families retrieved from lens.org. A more in-depth patent analysis was conducted separately (see Section 3.4).

Limitations include incomplete data for emerging products, and reliance on secondary sources for product stage determination and market size estimates. These factors may influence the accuracy of TRL classification and market potential estimates, underscoring the need for continuous updates and validation as new data becomes available.

#### 3.3.2. Wheel Visualization

**Figure 4:**
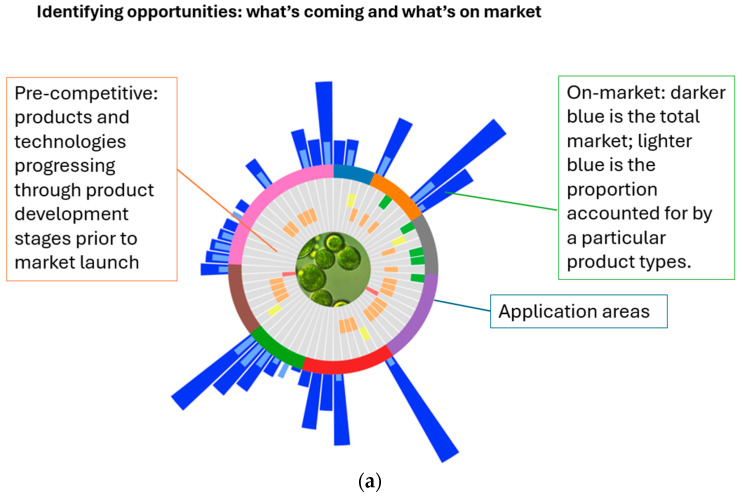

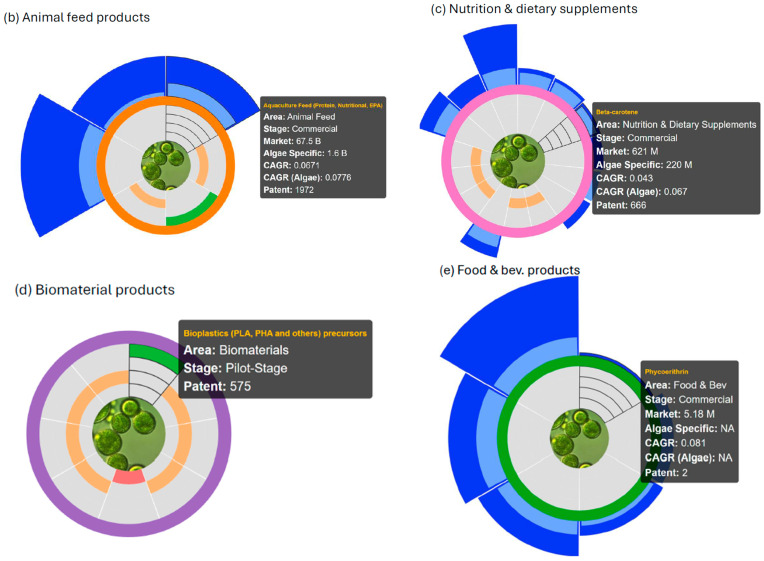
Product value-add opportunities wheels, with (**a**) the overall wheel configuration with inner gray bars representing the algae-based products, the colored bars their application area (e.g., food, animal feed, biofuel, etc.) and the outer blue bars their respective market sizes, with dark blue for total market, and light-blue for the specific algae-based fraction of the total market (where available). (**b**–**e**): Focused product value-add opportunities wheels by application areas: (**b**) animal feed products, (**c**) nutrition and dietary supplements, (**d**) biomaterials, and (**e**) food and beverage products, with highlights (gray box) for individual products (examples) of medicated animal feed, beta-carotene, bioplastics, and phycoerythrin, for each application area, respectively.

Based on the data sourced, a visual overview was developed: The Product Value-Add Opportunity Wheel, representing possible product development opportunities. It maps the product development stage by aligning the development level of products with their market size and growth for overall and microalgae-specific product markets. Figure 4a illustrates the overall product development landscape, while Figure 4b–e present examples across application categories. The inner-most ring represents the TRLs for each product, progressing from Fundamental Research, Applied Research, Pilot Stage, Pre-competitive, and Commercial stages. The outer bars indicate market size, with bar length corresponding to market value and the inner bar (light blue) showing the proportion attributable algal share of the market for the respective product.

As the Product Value Add Opportunity Wheel combines development stage and market size, the wheel can be used at multiple phases to support innovation, translation, and commercialization throughout the product development lifecycle:

- Ideation/Early-Stage Research (TRL 1–3): Researchers can quickly gauge whether a concept has translational potential by locating the product category on the wheel and checking (i) the development position of analogous algal products, (ii) the size and growth of the target market, and (iii) patent density (as a proxy for competitive intensity). A large market with modest patent activity could suggest opportunities; conversely, a small market with high patent activity flags potential saturation.
- Prototyping and Pilot (TRL 4–6): At this stage, teams can use the wheel to prioritize which prototypes advance to pilot by comparing market value bars with current TRLs and patent landscapes to estimate freedom-to-operate.
- Pre-Competitive to Commercial and Portfolio Strategy (TRL 7–9 and beyond): Product managers and executives can use the wheel’s combined TRL market signals to stage market entry strategies and balance their pipeline. By targeting categories where the algal share indicates room to grow and complementing this with competitive intelligence (e.g., product launches, claims, subcategory trends), they can refine positioning, prioritize near term revenue candidates, and plan for longer term “blockbuster” opportunities—mitigating cashflow risk during scaleup.

### 3.4. Patent Landscape Analysis

Patents are widely recognized as indicators of innovation and technological development [20]. They provide insights into the evolution of emerging technologies, competitive intensity, and areas of strategic opportunity. In the microalgae sector, patent activity reflects the race to unlock and protect novel solutions for cultivation, processing, and bioproduct extraction. Recent years have seen a significant increase in patent filings worldwide, underscoring growing commercial interest and the need for systematic intellectual property (IP) analysis to guide R&D and innovation commercialization strategies [21].

#### 3.4.1. Patent Analysis Methodology

Patent data were sourced from lens.org, focusing on single patent families to avoid duplication. Searches covered patents from the earliest recorded filings up to September 2025, providing a comprehensive view of historical and current trends. The search strategy combined microalgae-specific terms (e.g., Spirulina, microalgae, *Chlorella*) with product-specific terms (e.g., biofertilizer, aquaculture feed, omega-3), accounting for variations in spelling (British vs. American English) and alternative denominations for similar products.

Upon collating all related patents, analysis involved classification by species (e.g., *Chlorella*, Spirulina, *Nannochloropsis*), application domain (e.g., nutraceuticals, biofuels, animal feed), as well as temporal and special distributions. Contrary to the product wheel section, the patent analysis did take into consideration the environmental applications of microalgae, which are covered under the section “process applications”. The patent analysis was conducted on a global scale, enabling identification of technological clusters, key patent holders, and emerging innovation spaces.

#### 3.4.2. Patent Analysis Visualization

**Figure 5:**
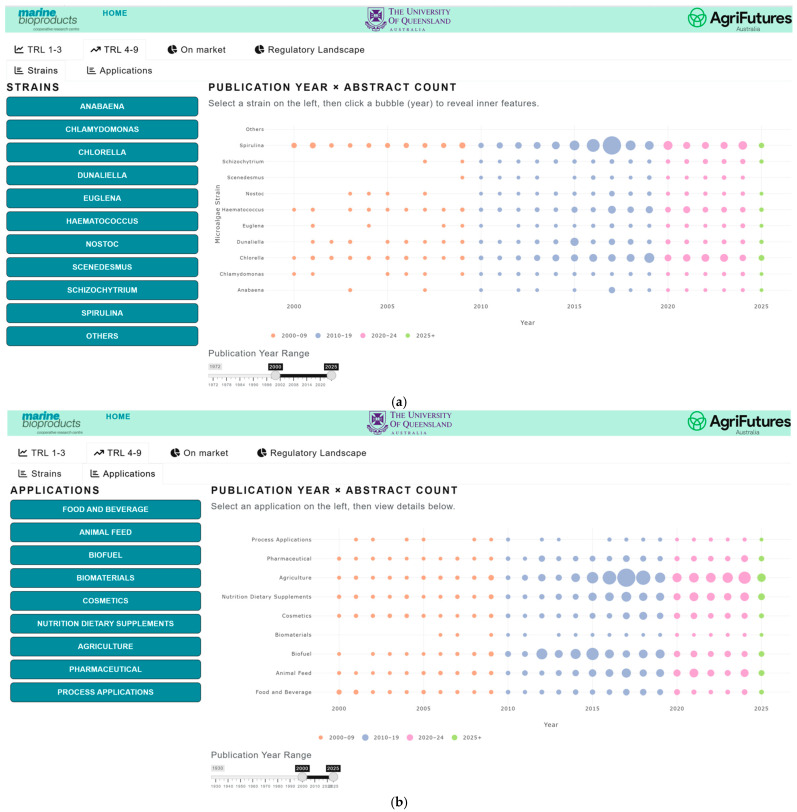
Microalgae Patent landscape, showing the number of patent publications per year between 2000 and 2025 for (**a**) the top 10 most patented microalgae species, and (**b**) microalgae-product applications areas, with larger circles indicating more patents.

**Figure 6:**
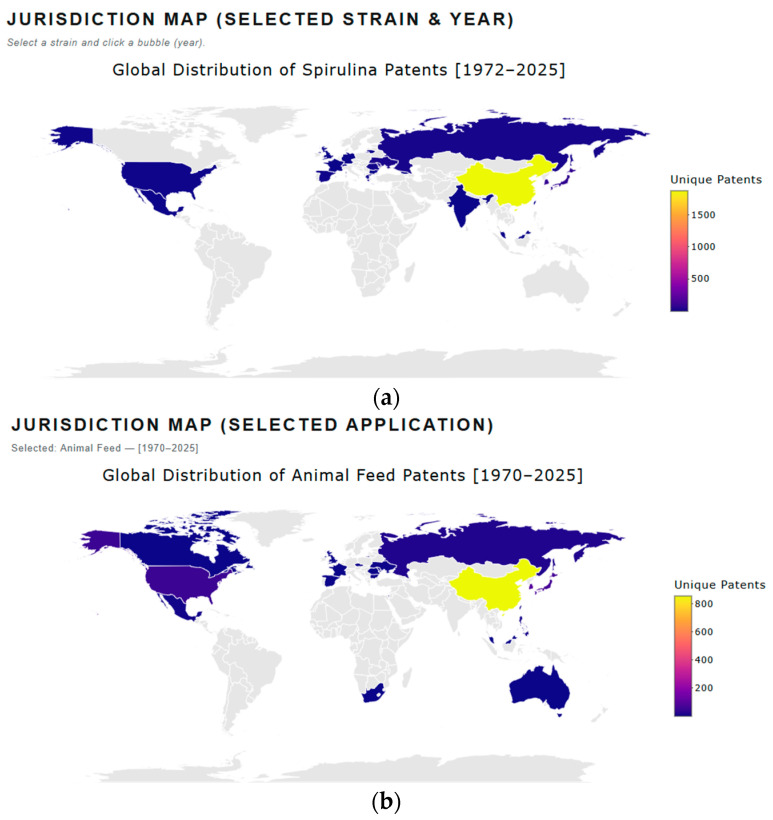
(**a**,**b**): Patent by jurisdiction.

The patent landscape is presented as a heat map, providing a longitudinal overview of areas with intense patent activity (“hot spots”) and regions with minimal filings (“white spaces”) by species (Figure 5a) and applications (Figure 5b). Hot spots indicate domains with strong IP protection activity, while white spaces can indicate areas which are not very attractive, where previous efforts have failed or revealed underexplored areas that may represent potential opportunities for future innovation and research [22]. The interactive dashboard allows users to drill down into specific patent clusters by clicking on a group to view the underlying patents. Additionally, users can visualize the spatial distribution of patent filings over a defined time period, enabling analysis of geographic trends (Figure 6) and temporal dynamics in technological development.

From a competitive intelligence perspective, the patent landscape serves multiple purposes. It enables identification of saturation and whitespace, helping firms identify crowded intellectual property spaces and target possible niches. It also signals emerging areas with low patent density but high market potential, which represent opportunities for strategic innovation. Furthermore, patent clustering assists in assessing freedom-to-operate, allowing companies to evaluate intellectual property risks before scaling up. Finally, understanding patent holders and filing trends provides a basis for benchmarking competitors and informing partnership or licensing opportunities. The observed increase in patent applications underscores the importance of integrating patent analytics into early-stage product development and commercialization planning.

### 3.5. Market and Competitive Landscape

The market and competitive landscape analysis provides insights into market size, growth trends, and competitive intensity for microalgae-based products. By mapping product launches, claims, and sub-category trends, this section helps businesses understand serviceable market opportunities and potential threats, supporting strategic decision-making for commercialization.

#### 3.5.1. Methodology

The market analysis is built from a range of sub-analyses of data related to the volume, value (manufacturing as well as retail), company and brand share, and distribution channel, sourced from Euromonitor database. The analysis incorporates historical and current data to produce temporal and spatial assessments with some forecasting. However, one of the main limitations is the availability of time series algae/microalgae specific market data. Therefore, key market analysis of the major application areas (e.g., pet food, skin care, natural colors, carotenoids, carrageenans, dietary supplements) are presented. The product launches and claims data were sourced from Mintel datasets rather than reports. Searches covered product launches from July 1996 up to August 2025, providing a comprehensive view of historical and current trends. The same search strategy of microalgae-specific terms (e.g., Spirulina, microalgae, *Chlorella*), as performed in patent analysis, was followed. Large Language Models (LLM) were used for product claims classification.

#### 3.5.2. Visualization

**Figure 7:**
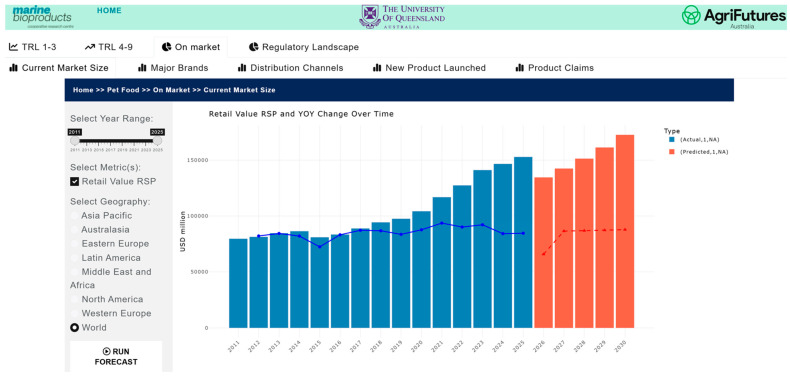
Pet food market size—actual (blue) and predicted (orange) trends in value over 2011–2030.

**Figure 8:**
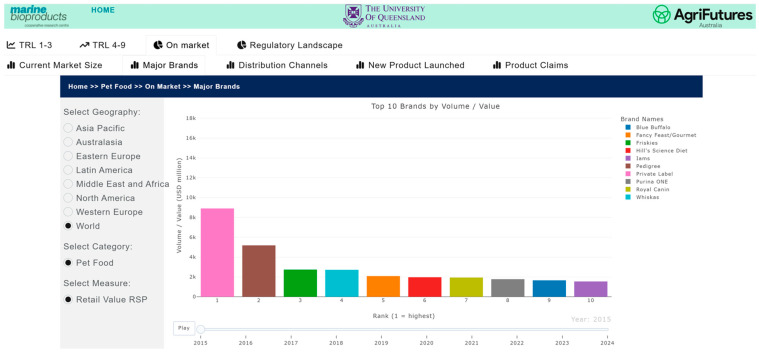
Major brands by value.

**Figure 9:**
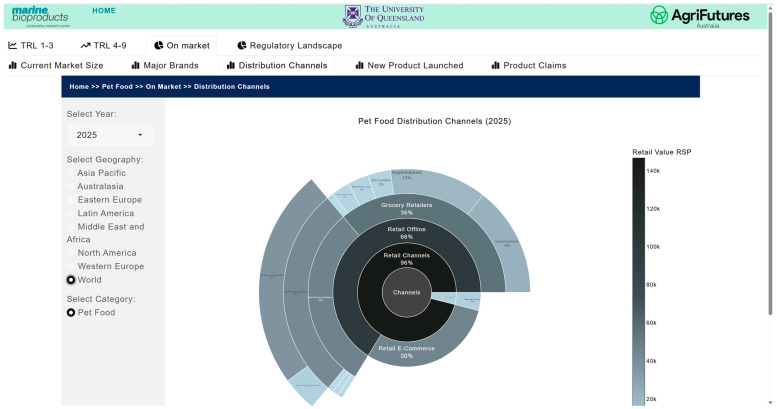
Pet food distribution channel (2025).

**Figure 10:**
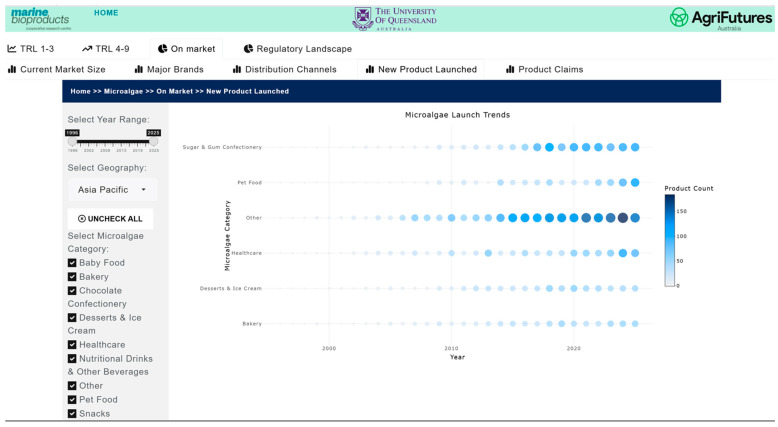
Product launches in the food, beverage, and healthcare sectors with microalgae ingredients.

**Figure 11:**
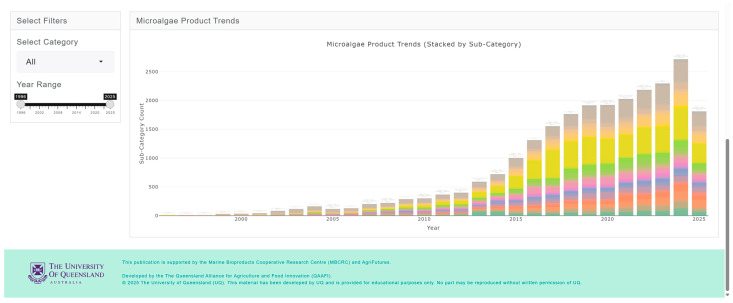
Product launches with microalgae ingredients by sub-categories, represented by different colors in each of the bars.

**Figure 12:**
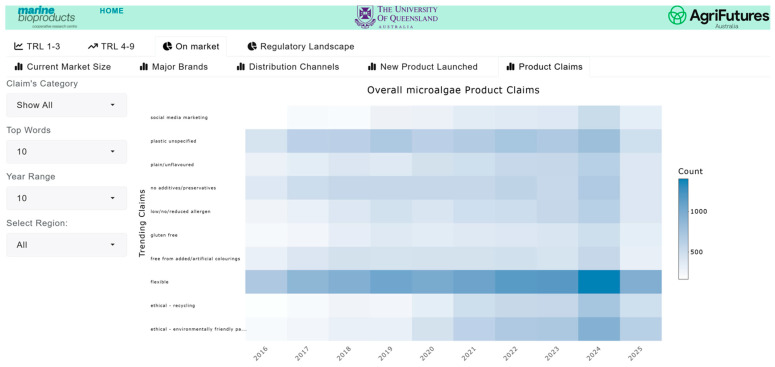
Temporal distribution of top product claims in products with microalgae ingredients.

The market and competitive landscape are presented through historical data and trends in market size, major companies and brands, distribution channels, product launches, and major claims. Figure 7 shows a case example of the trend in markets and its growth for pet food. The market landscape also shows major brands (Figure 8) and distribution channels (Figure 9), as well as product launches in food, beverage and healthcare (Figure 10) and its sub-categories (Figure 11) and claims (Figure 12) for products with microalgae as one of their ingredients. Capturing and analyzing the demand/market pull dynamics facing a new product, technology or service as it progresses through its development cycle is essential for finding a realistic market niche that will support market entry and penetration.

The integration of market and competitive analytics into planning enables businesses to identify market opportunities and gaps that signal greater freedom to operate. Insights into consumer trends and product claims allow companies to align positioning with evolving preferences, strengthening brand relevance and market acceptance.

### 3.6. Navigating Regulatory Pathways

Regulatory compliance is a critical determinant of market access for microalgae-based products. Complex and evolving regulations govern activities across the value chain—from strain collection and cultivation to processing, product development, and commercialization. Failure to anticipate these requirements early can lead to costly delays, market entry obstacles and potential failures. To address this challenge, the dashboard integrates a regulatory guide and interactive navigation tool designed to help businesses and researchers systematically identify compliance obligations and optimize their regulatory pathway. This feature complements technical and market analytics, ensuring that innovation strategies are aligned with legal requirements from the outset.

#### 3.6.1. Methodology

The regulatory guide was developed through an extensive review of Australian legislation and standards at both state and national levels, supplemented by international requirements relevant to trade and export. Key regulatory junctures were mapped along the microalgae value chain, including strain collection and import (e.g., biodiversity access, biosecurity permits), research and development (e.g., GMO containment, dual-use technologies, legal and ethical considerations), commercial cultivation (e.g., production licenses, environmental approvals, water use licenses), and product development and commercialization (e.g., TGA, FSANZ, export permits).

Data sources included statutory instruments, regulatory agency guidelines, and industry codes of practice. These were synthesized into a regulatory pathways’ navigation tool and compliance checkpoints, enabling users to navigate obligations based on their operational context (e.g., strain type, cultivation method, intended product category, and market destination).

#### 3.6.2. Visualization

**Figure 13:**
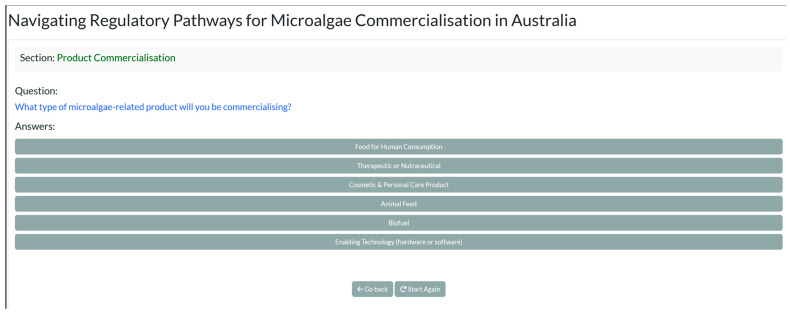
Tool for navigating the regulatory pathways for microalgae commercialization.

The regulatory component is presented in two formats: an interactive Regulatory Pathway Navigation Tool (Figure 13) where users respond to a series of prompts (e.g., strain origin, GMO status, intended application) to generate a tailored compliance pathway. This dynamic tool highlights required permits, approvals, and standards at each stage specific to a user’s scenario. It is accompanied by a Regulatory Overview Report—a structured overview of applicable regulations, including links to official guidelines and agencies, providing a reference for detailed compliance planning.

Both formats are integrated into the dashboard, allowing seamless navigation between technical readiness, market analytics, and regulatory requirements.

The regulatory navigation tool supports strategic decision-making across multiple phases:

- Early R&D: Researchers can identify whether strain collection requires biodiversity permits or biosecurity clearance before initiating experiments.
- Scale-Up Planning: Businesses and entrepreneurs can anticipate environmental approvals and cultivation licenses needed for commercial production.
- Product Development: New product development teams can verify food safety, therapeutic classification, and GMP obligations for downstream processing.
- Market Entry: Export-oriented firms can assess Codex compliance, customs documentation, and international trade requirements.

For example, Figure S1 illustrates the regulatory pathway for an Australian business planning to commercially cultivate a non-GMO native microalga strain from a third party, in a land-based cultivation system, and produce microalgae-based animal feed classified as an Excluded Nutritional or Digestive (END) product for exports. Figure S2 shows the entire nodes for the whole regulatory navigation pathways.

## 4. Discussion

Commercializing microalgae products demands integrative development and strategic alignment in technical maturity, regulatory compliance, and market positioning. The dashboard addresses this complexity by incorporating key pre-market and market dimensions—technological, markets and product trends, and competitive landscape—with TRLs, market analytics, IP signals, and regulatory pathways in a unified decision-support system. This integration enables firms to align technical development with consumer demand and industry standards, moving beyond siloed analyses toward coordinated strategies that reduce risk, accelerate scale-up, and guide strategic investment and commercialization.

Key integration elements of the dashboard:

- IP analytics for opportunity selection: Patent landscape analysis complements the Product Value-Add Opportunity Wheel by revealing saturated domains and opportunities across microalgae species and applications. Together, these insights guide product selection, differentiation strategies, and licensing decisions, while interactive drilldowns add granularity for freedom-to-operate assessments.
- Linking product maturity to market dynamics: TRLs are coupled with market intelligence—size, growth, product launches, and claims—to prioritize innovations that are both technically feasible and commercially attractive. The Product Readiness Assessment tool reinforces this by providing structured evaluations technical, market, financial, and regulatory readiness across the innovation lifecycle.
- Regulatory navigation embedded in analytics: The dashboard embeds regulatory pathways within the same environment that tracks technology readiness, market signals and IP positioning. The interactive tool dynamically links regulatory requirements to product development stages and market entry strategies, generating scenario-specific pathways that reflect technical maturity and competitive context. This integration aligns compliance planning with investment priorities and innovation timelines, reducing late-stage surprises and clarifying approval sequences.

Beyond individual tools, the dashboard supports portfolio strategies that balance near-term revenue products with longer-term “blockbusters,” mitigating cash-flow risks that often cause failure during high-expenditure or early growth phases. By embedding regulatory, market, and technical analytics in an integrated system, it enables firms to set priorities, track progress, and make evidence-informed decisions about engagement with emerging technologies—whether operating locally or globally.

Because the dashboard integrates analytics from multiple sources within an emerging industry, limited data availability remains a significant challenge. This constraint is compounded by the rapidly evolving nature of legislation, policies, and political conditions, which can trigger swift changes in both market and regulatory environments. The dashboard development process highlighted the importance of interdisciplinary expertise and iterative co-design, incorporating stakeholder feedback across phases to ensure relevance and usability. Similar collaborative approaches have been emphasized in other domains, such as clinical dashboard development [23,24]. To remain effective amid dynamic trends and shifting market conditions, the dashboard must be designed for adaptability and continuous updates. Artificial intelligence (AI), under robust governance and ethical oversight, offers a pathway to enhance functionality through interactive analytics, context-rich insights, and adaptive visual interfaces tailored to user roles and interaction patterns [25].

## 5. Conclusions

While microalgal industry holds transformative potential, their realization as a cornerstone of the global bioeconomy depends on overcoming significant technological and economic hurdles through sustained research, innovation, and supportive regulatory frameworks. The framework presented offers an integrated approach by combining multiple analytical components—product readiness assessment, pre-competitive, and competitive market analytics and regulatory pathway navigation. The dashboard presented moves beyond fragmented analyses by combining economics, market, innovation, and strategy theories that provide a comprehensive and integrated analytical framework for understanding the technology development, market, and competitive landscape of the microalgae industry. It provides strategic approaches to navigating complex landscapes of microalgae-based product development by integrating technical maturation, regulatory compliance, and market readiness into a unified model, offering a strategic tool for researchers, developers, and policymakers. The novelty of theF framework lies in its holistic integration of disparate but interdependent domains that are typically analyzed in isolation.

In terms of the limitations, its outputs are constrained by the availability, granularity, and currency of microalgae-specific market and regulatory data, which remain fragmented and inconsistent across jurisdictions. Despite the limitations, the knowledge and tools provided through the dashboard could help industry partners target key markets, optimize systems, processes and value chains to ensure that they meet market requirements and comply with the necessary regulations. Looking ahead, the dashboard offers a foundation for adaptive analytics that can evolve with emerging data, shifting regulations, and global market trends. AI-powered enhancements and stakeholder co-design will be critical to maintaining relevance and usability, ensuring that the system remains a living resource rather than a static model. While focused on the microalgal sector, the principles underpinning this framework have broader applicability across marine bioproducts and other bio-based industries, supporting the transition toward a resilient and sustainable bioeconomy.

## Data Availability

The datasets presented in this article are proprietary and not readily available.

## Supplementary Materials

The following supporting information can be downloaded at: https://www.mdpi.com/article/10.3390/md24020066/s1, Table S1: Details for the Product Opportunity Wheel development; Figure S1: Regulatory pathway for commercial production and export of microalgae END feed; Figure S2: Full regulatory pathway nodes for microalgae in Australia.

## Abbreviations

The following abbreviations are used in this manuscript:

AI	Artificial Intelligence
CAGR	Compound Annual Growth Rate
LLMs	Large Language Models
NASA	National Aeronautics and Space Administration
R&D	Research and Development
TRL	Technology Readiness Level
