# Supplementary material for: Analytical Framework to Navigate Microalgae-Based Product Development—Aligning Commercialization and Regulatory Pathways
Source: Mar Drugs. 2026 Feb 3;24(2):66. doi: 10.3390/md24020066 (PMC12941414; doi:10.3390/md24020066)
Supplement: Supplementary file 1 [file marinedrugs-24-00066-s001.zip › Table S1-Details for Product Opportunity Wheel development.pdf]

Appendix A.3: Wheel visualization

Product	Primary Application Area	Development Stage	Market Size (US$)	CAGR	Algae Specific Market (if available) (US$)	CAGR (Algae Specific Market)	Source
Biofertilisers and Biostimulants	Agriculture	Commercial	4.03 B (2024)<br>2.6 B (2022)	11.80% (2025-2032)<br>7.4% (2022-2030)	13.64 B (2024)<br>13.3 B (2025)<br>748.55 M (2025)	9.88% (2025-2034)<br>9.5% (2025-2035)<br>7.51 % (2025-2030)	https://www.sciencedirect.com/science/article/pii/S2352186425004663<br>https://www.fortunebusinessinsights.com/industry-reports/biostimulants-market-100414<br>https://www.grandviewresearch.com/industry-analysis/biostimulants-market<br>https://royalsocietypublishing.org/doi/pdf/10.1098/rstb.2024.0251<br>https://www.precedenceresearch.com/microalgae-fertilizers-market<br>https://www.futuremarketinsights.com/reports/microalgae-fertilizers-sector<br>https://www.researchandmarkets.com/report/algae-fertilizer?srsltid=AfmBOoqOG7GfuMP5aE9CP_SQ_A4ozUckjlvQultr8YpjpXknTv62NVx6
Biopesticides	Agriculture	Pre-competitive	7.72 B (2024)<br>8.73 B (2024)	15.2% (2024-2029)<br>16% (2025-2032)	n/a	n/a	https://cinea.ec.europa.eu/featured-projects/algaenauts-sustainable-biopesticides-based-microalgae_en<br>https://www.algaenauts.eu/wp-content/uploads/2024/04/D5.4-Marketability-market-analysis-on-target-products.pdf<br>https://www.marketsandmarkets.com/Market-Reports/biopesticides-267.html<br>https://www.fortunebusinessinsights.com/industry-reports/biopesticides-market-100073<br>https://ag.algaenergy.com/bio-control/
Biochar	Agriculture	Applied Research	763.48 M (2024)	13.6% (2025-2032)	n/a	n/a	https://link.springer.com/article/10.1007/s10098-025-03192-y<br>https://www.fortunebusinessinsights.com/industry-reports/biochar-market-100750<br>https://biochar-international.org/wp-content/uploads/2024/06/Global-Biochar-Market-Report-2023-—-Public.pdf
Aquaculture Feed (Protein, Nutritional, EPA)	Animal Feed (incl. Aquaculture)	Commercial	72.5 B (2022)<br>67.5 B (2024)	4.4% (2023-2030)<br>6.71% (2025-2032)	276.23 M (2024)<br>27.5% of microalgae animal feed market (4.26B) 1.17 B (2025)<br>1.6 B (2024)	7.76% (2025-2032)	https://link.springer.com/article/10.1007/s11356-021-14989-x<br>https://www.fortunebusinessinsights.com/industry-reports/microalgae-based-aquafeed-market-100494<br>https://www.grandviewresearch.com/industry-analysis/aquafeed-and-aquaculture-additive-market<br>https://www.fortunebusinessinsights.com/industry-reports/aquafeed-market-100698<br>https://www.futuremarketinsights.com/reports/algae-based-animal-feed-market<br>https://www.gminsights.com/industry-analysis/algae-based-animal-feed-market
Medicated Animal Feed	Animal Feed (incl. Aquaculture)	Applied Research	4.01 B (2024)<br>12.72 B (2025)	5.2% (2024-2032)<br>5.66% (2025-2030)	n/a	n/a	https://www.sciencedirect.com/science/article/pii/S0048969722047553<br>https://www.fortunebusinessinsights.com/medicated-feed-additives-market-113276<br>https://www.mordorintelligence.com/industry-reports/medicated-feed-additives-market
Poultry Feed	Animal Feed (incl. Aquaculture)	Pilot-Stage	205.44 B (2022)	4.6% (2022-2030)	Estimated at 34% of microalgal animal feed market (4.26B)<br>1.44 B (2025)	n/a	https://www.frontiersin.org/journals/veterinary-science/articles/10.3389/fvets.2024.1382163/pdf<br>https://www.sciencedirect.com/science/article/pii/S221192642400345X<br>https://www.grandviewresearch.com/horizon/outlook/poultry-feed-market-size/global<br>https://www.futuremarketinsights.com/reports/algae-based-animal-feed-market
Cattle Feed	Animal Feed (incl. Aquaculture)	Applied Research	133.5 B (2025)	3.8% (2025-2035)	n/a	n/a	https://www.futuremarketinsights.com/reports/cattle-feed-market
Animal Feed (overall)	Animal Feed (incl. Aquaculture)	Commercial	570.72 B (2022)	4.6% (2022-2030)	4.5 B (2024)<br>4.26 B (2025)	3.8 % (2024-2034)<br>4.2 % (2025-2035)	https://www.gminsights.com/industry-analysis/algae-based-animal-feed-market<br>https://www.grandviewresearch.com/industry-analysis/animal-feed-market-report<br>https://www.futuremarketinsights.com/reports/algae-based-animal-feed-market
Pet Feed	Animal Feed (incl. Aquaculture)	Commercial	103.3 B (2023)<br>34.2 B (2023)	4.4% (2023-2030)<br>6.8% (2023-2028)	25.5 M (2025)	4.8% (2025-2035)	https://www.futuremarketinsights.com/reports/microalgae-pet-food-sector<br>https://www.marketsandmarkets.com/Market-Reports/global-pet-food-and-care-products-market-147.html<br>https://www.grandviewresearch.com/industry-analysis/pet-food-industry
Biodiesel	Biofuels	Pilot-Stage	32.09 B (2021)<br>57.91 B (2024)	10% (2022-2030)<br>6.2% (2025-2029)	n/a	n/a	https://www.mdpi.com/2076-2607/11/1/34<br>https://www.grandviewresearch.com/industry-analysis/biodiesel-market<br>https://www.researchandmarkets.com/reports/5782777/biodiesel-global-market-<br>report?utm_source=GNE&utm_medium=PressRelease&utm_code=n7j96m&utm_campaign=2041824+-<br>+Analyzing+the+Potential+%2477.94+Billion+Biodiesel+Market%2c+2025-2029+%26+2034&utm_exec=jocamspi
Biofuels (Overall)	Biofuels	Pre-competitive	99.53 B (2023)<br>160.56 B (2024)	11.3% (2024-2030)<br>6.84 % (2025-2032)	8.38 B (2022)	10.1% (2023-2030)	https://www.grandviewresearch.com/industry-analysis/algae-biofuel-market<br>https://www.grandviewresearch.com/industry-analysis/biofuels-market<br>https://www.fortunebusinessinsights.com/liquid-biofuels-market-103294<br>https://www.sciencedirect.com/science/article/pii/S0961953425005033
Biogas	Biofuels	Applied Research	140.89 B (2025)<br>65.53 B (2023)	4.46% (2025 -2032)<br>4.2% (2024-2030)	n/a	n/a	https://biointerfaceresearch.com/wp-content/uploads/2021/08/20695837123.38493882.pdf<br>https://www.fortunebusinessinsights.com/industry-reports/biogas-market-100910<br>https://www.grandviewresearch.com/industry-analysis/biogas-market
Bioethanol	Biofuels	Pilot-Stage	75.96 B (2023)	5.33% (2024-2032)	n/a	n/a	https://link.springer.com/chapter/10.1007/978-3-031-85300-5_18<br>https://www.fortunebusinessinsights.com/industry-reports/bioethanol-market-101076
Biocrude oil	Biofuels	Pilot-Stage	n/a	n/a	n/a	n/a	https://link.springer.com/chapter/10.1007/978-981-96-5198-6_8
Biohydrogen (green Hydrogen)	Biofuels	Applied Research	7.98 B (2024)	38.5% (2025-2030)	n/a	n/a	https://link.springer.com/chapter/10.1007/978-3-031-75132-5_1<br>https://www.grandviewresearch.com/industry-analysis/green-hydrogen-market
Bioplastics (PLA, PHA and others) precursors	Biomaterials	Pilot-Stage	15.57 B (2024)	19.5% (2025-2030)	106.2 M (2025)	5.2 (2025-2034)	https://link.springer.com/chapter/10.1007/978-981-96-5198-6_9<br>https://www.gminsights.com/industry-analysis/algae-based-plastics-market<br>https://www.businessresearchinsights.com/market-reports/polylactic-acid-pla-bioplastic-market-125568<br>https://www.grandviewresearch.com/industry-analysis/bioplastics-industry
Hydrogels	Biomaterials	Applied Research	34.25 B (2024)<br>31.96 B (2025)	7.4% (2025-2030)<br>6.7% (2025-2034)	n/a	n/a	Hydrogel Market Size And Share | Industry Report, 2030<br>https://www.sciencedirect.com/science/article/pii/S2352940725002756<br>https://www.precedenceresearch.com/hydrogel-market
Nanocellulose	Biomaterials	Applied Research	351.5 M (2022)<br>600 M (2024)	20.1% (2023-2030)<br>23.7% (2024-2032)	n/a	n/a	https://www.grandviewresearch.com/industry-analysis/nanocellulose-market<br>https://www.marketsandmarkets.com/Market-Reports/nano-cellulose-market-56392090.html
Biodegradable films	Biomaterials	Applied Research	1.3 B (2024)	7.7% (2025-2034)	n/a	n/a	https://link.springer.com/chapter/10.1007/978-981-96-5198-6_9<br>https://www.gminsights.com/industry-analysis/biodegradable-films-market
Protein-based adhesives	Biomaterials	Fundamental Research	2.4 B (2024)<br>1.18 B (2024)	6.5% (2024-2034)<br>6.4% (2024-2033)	n/a	n/a	https://growthmarketreports.com/report/protein-based-adhesives-market<br>https://www.factmr.com/report/protein-glue-market
Activated Charcoal	Biomaterials	Applied Research	3.9 B (2024)<br>5.7 B (2024)	3.8% (2025-2033)<br>8.7% (2025-2032)	n/a	n/a	https://www.mdpi.com/2624-7402/1/2/14<br>https://www.grandviewresearch.com/industry-analysis/activated-carbon-market<br>https://www.fortunebusinessinsights.com/activated-carbon-market-102175
Graphene	Biomaterials	Applied Research	694 M (2024)<br>195.7 M (2023)	36.5% (2025-2032)<br>35.1% (2024-2030)	n/a	n/a	https://www.fortunebusinessinsights.com/graphene-market-102930<br>https://www.grandviewresearch.com/industry-analysis/graphene-industry
Nano Carbon	Biomaterials	Applied Research	5.83 B (2024)	27.5% (2024-2034)	n/a	n/a	https://biotechnologyforbiofuels.biomedcentral.com/articles/10.1186/s13068-019-1465-0<br>https://www.precedenceresearch.com/carbon-nanomaterials-market

EPS (Extracellular Polymeric Substances)	Biomaterials	Applied Research	8.5 B (2024)	5.2% (2023-2032)	n/a	n/a	https://www.mdpi.com/1996-1073/14/13/4007<br>https://dataintelo.com/report/global-extracellular-polymeric-substances-market
Personal Care and Cosmetics (general)	Cosmetics	Commercial	557 B (2023)<br>605.23 B (2025)	7.7% (2024-2030)<br>5.11% (2025-2030)	64.1 M (2024)<br>68.5 M (2025)	3.7% (2024-2031)<br>5.5% (2025-2035)	https://chemicalresearchinsight.com/2025/08/05/top-10-companies-in-the-microalgae-for-cosmetics-industry-2025-market-leaders-powering-clean-beauty-innovations/<br>https://www.futuremarketinsights.com/reports/microalgae-personal-care-and-cosmetics-sector<br>https://www.grandviewresearch.com/industry-analysis/beauty-personal-care-products-market<br>https://www.mordorintelligence.com/industry-reports/global-beauty-and-personal-care-products-market-industry
Squalene	Cosmetics	Pre-competitive	149.4 M (2023)	10.9% (2024-2030	n/a	n/a	https://link.springer.com/article/10.1007/s12257-021-0355-z<br>https://www.grandviewresearch.com/industry-analysis/squalene-market
Scytonemin (UV Absorbing)	Cosmetics	Applied Research	n/a	n/a	n/a	n/a	https://www.mdpi.com/1660-3397/19/3/129
Cosmetic thickeners	Cosmetics	Applied Research	3.8 B (2024)	7.5% (2024-2032)	n/a	n/a	https://link.springer.com/chapter/10.1007/978-3-030-50971-2_25<br>https://dataintelo.com/report/global-cosmetic-thickener-market
Lipid-based moisturizers	Cosmetics	Applied Research	10 B (2022)<br>3.42 B (2024)	5.5% (2023-2032)<br>4.02% (2025-2035)	n/a	n/a	https://www.mdpi.com/2079-9284/8/2/52<br>https://www.gminsights.com/industry-analysis/cosmetic-lipid-ingredients-market<br>https://www.marketresearchfuture.com/reports/cosmetic-lipid-ingredient-market-33891
Skin-Care Ingredients	Cosmetics	Commercial	115.65 B (2024)	6.84 % (2024-2032)	Approx. 40 M (2023)	7.2% (2024-2030)	https://www.grandviewresearch.com/industry-analysis/algae-skincare-products-market-report<br>https://www.fortunebusinessinsights.com/skin-care-market-102544
Sun Care Products	Cosmetics	Commercial	12.44 B (2024)	4.2% (2025-2030)	n/a	n/a	https://onlinelibrary.wiley.com/doi/pdf/10.1111/ics.13019<br>https://www.grandviewresearch.com/industry-analysis/sun-care-products-market-report
Anti-aging formulations	Cosmetics	Commercial	52.44 B (2024)	7.7% (2025-2030)	n/a	n/a	https://link.springer.com/article/10.1007/s10811-024-03345-4<br>https://www.grandviewresearch.com/industry-analysis/anti-aging-products-market
Natural cosmetic colorants	Cosmetics	Commercial	737.45 M (2023)	7.4% (2024-2030)	n/a	n/a	https://www.mdpi.com/2079-9284/9/1/10<br>https://www.grandviewresearch.com/industry-analysis/cosmetic-pigments-market-report
Phycoerithrin	Food & Beverages	Commercial	42.3 M (2023)<br>5.18 M (2025)	7.2% (2024-2033)<br>8.1% (2024-2035)	n/a	n/a	https://link.springer.com/article/10.1007/s10811-022-02834-8<br>https://www.grandviewresearch.com/industry-analysis/natural-food-colors-market<br>https://www.futuremarketinsights.com/reports/phycoerythrin-market
Microalgae based food (overall)	Food & Beverages	Commercial			670.45 M (2024)	8.61% (2024-2032)	https://www.fortunebusinessinsights.com/industry-reports/microalgae-food-market-100802
Pigments & Natural food colorants	Food & Beverages	Commercial	1.32 B (2022)	8.3% (2023-2030)	26 M (2025)	7.4% (2025-2035)	https://link.springer.com/chapter/10.1007/978-981-15-0169-2_3<br>https://www.grandviewresearch.com/industry-analysis/natural-food-colors-market<br>https://www.futuremarketinsights.com/reports/algal-pigments-market
Algal protein powder	Food & Beverages	Commercial	24.6 B (2024)	6.8% (2024-2034)	997.4M (2024)	4.8% (2025-2030)	https://academic.oup.com/ijfst/article/59/1/473/7808034<br>https://www.grandviewresearch.com/industry-analysis/algae-protein-market<br>https://www.gminsights.com/industry-analysis/protein-powder-market
Functional food ingredients	Food & Beverages	Commercial	73.1 B (2022)	6.6% (2023-2030)	4.24 B (2024)	7.5% (2025-2034)	https://pmc.ncbi.nlm.nih.gov/articles/PMC9818788/<br>https://www.insightaceanalytic.com/report/global-algae-based-ingredients-market/1461<br>https://www.grandviewresearch.com/industry-analysis/functional-ingredients-market
Wastewater Treatment	Industrial & Chemical Products	Commercial	371 B (2025)	6.55% (2025-2034)	2.65 B (2024)	5.89% (2025-2032)	https://www.frontiersin.org/journals/analytical-science/articles/10.3389/frans.2024.1513153/full<br>https://www.towardschemandmaterials.com/insights/water-and-wastewater-treatment-market<br>https://www.datamintelligence.com/research-report/algae-based-wastewater-treatment-market
Carbon Capture	Industrial & Chemical Products	Pre-competitive	4.51 B (2025)	18.18% (2025-2032)	n/a	n/a	https://www.sciencedirect.com/science/article/abs/pii/S0013935124003013<br>https://www.fortunebusinessinsights.com/industry-reports/carbon-capture-and-sequestration-market-100819
Fermentation substrates	Industrial & Chemical Products	Applied Research	75 B (2023)	6.9% (2024-2030)	n/a	n/a	https://www.mdpi.com/2076-2607/10/10/2069<br>https://www.grandviewresearch.com/industry-analysis/fermentation-chemicals-market
Biolubricants	Industrial & Chemical Products	Applied Research	2.95 B (2024)	13.7% (2025-2030)	n/a	n/a	https://www.sciencedirect.com/science/article/pii/S004896972406916X<br>https://www.grandviewresearch.com/industry-analysis/biolubricants-industry
Biosurfactants and emulsifiers	Industrial & Chemical Products	Applied Research	4.41 B (2023)	5.4% (2024-2032)	n/a	n/a	https://research.tees.ac.uk/en/publications/biosurfactants-and-bio-emulsifiers-from-algae-3<br>https://www.fortunebusinessinsights.com/biosurfactants-market-102761
Enzyme production substrates	Industrial & Chemical Products	Applied Research	13.9 B (2024)	6.6% (2025-2033)	n/a	n/a	https://link.springer.com/chapter/10.1007/978-3-031-86433-9_19<br>https://www.grandviewresearch.com/industry-analysis/enzymes-industry
Fluorescent dyes	Industrial & Chemical Products	Fundamental Research	377 M (2023)	5.9% (2024-2040)	n/a	n/a	https://link.springer.com/protocol/10.1007/7651_2017_101<br>https://www.grandviewresearch.com/industry-analysis/fluorescent-pigment-market-report
Natural textile dyes	Industrial & Chemical Products	Commercial	4.8 B (2024)	5.7% (2024-2029)	128.4 M (2024)	14.3% (2025-2033)	https://link.springer.com/article/10.1007/s40726-025-00363-9<br>https://www.marketsandmarkets.com/Market-Reports/natural-dyes-market-78173284.html<br>https://dataintelo.com/report/algae-based-textile-dye-market
Omega-3 fatty acids	Nutrition & Dietary Supplements	Commercial	2.62 B (2023)	7.9% (2024-2030)	850 M (2024)	7.4% (2024-2032)	Omega 3 Market Size, Share & Growth Analysis Report, 2030<br>https://finance.yahoo.com/news/algae-omega-3-market-reach-153000081.html
Astaxanthin	Nutrition & Dietary Supplements	Commercial	1.69 B (2024)	17.47% (2025-2030)	722.7 (2024)	18.7% (2024-2030)	https://www.grandviewresearch.com/horizon/statistics/astaxanthin-market/natural/microalgae/global
Beta-carotene	Nutrition & Dietary Supplements	Commercial	1.03 B (2023)<br>621 M (2024)	4.3% (2024-2030)	401 M (2023)<br>220 M (2024)	6.7% (2024-2033)	https://www.grandviewresearch.com/industry-analysis/beta-carotene-market<br>https://pdf.marketpublishers.com/grand/beta-carotene-market-analysis-by-source.pdf<br>https://marketintelo.com/report/algae-beta-carotene-market
Lutein	Nutrition & Dietary Supplements	Commercial	357.5 M (2023)<br>381.1 M (2024)	5.8% (2024-2030)<br>6.8% (2025-2035)	n/a	n/a	https://www.sciencedirect.com/science/article/pii/S2352186423000937<br>https://www.factmr.com/report/1075/lutein-market<br>https://www.grandviewresearch.com/industry-analysis/lutein-market-report
Phycocyanin	Nutrition & Dietary Supplements	Commercial			175.4 M (2023)	6.9% (2023-2040)	https://www.mdpi.com/2071-1050/17/13/5962<br>https://www.grandviewresearch.com/industry-analysis/phycocyanin-market-report
Fucoxanthin	Nutrition & Dietary Supplements	Commercial	219 M (2024)<br>209.45 M (2024)	5% (2025-2032)<br>5% (2025-2030)	n/a	n/a	https://virtuemarketresearch.com/report/fucoxanthin-market<br>https://www.maximizemarketresearch.com/market-report/global-fucoxanthin-market/98879/
Phytosterols	Nutrition & Dietary Supplements	Applied Research	1.06 B (2024)<br>0.92 B (2024)	9.4% (2025-2030)<br>9.1 % (2025-2035)	n/a	n/a	https://www.futuremarketinsights.com/reports/phytosterols-market<br>https://www.grandviewresearch.com/industry-analysis/phytosterols-market<br>https://www.sciencedirect.com/science/article/pii/S2211926419306836
Chlorophyllin (Cu)	Nutrition & Dietary Supplements	Applied Research	50 M (2023)<br>255 M (2025)	5.1% (2023-2032)<br>5.7% (2025-2033)	n/a	n/a	https://www.cognitivemarketresearch.com/chlorophyllin-market-report<br>https://dataintelo.com/report/global-chlorophyllin-market
Prebiotic dietary fibers	Nutrition & Dietary Supplements	Commercial	7.1 B (2025)	6.3% (2025-2035)	872 M (2024)	10.6% (2021-2024)	https://www.sciencedirect.com/science/article/pii/S1756464623002036<br>https://www.futuremarketinsights.com/reports/prebiotic-fiber-market<br>https://dataintelo.com/report/algal-prebiotic-fibers-market

Glycerol	Nutrition & Dietary Supplements	Applied Research	5.6 B (2024)	11.9% (2025-2034)	n/a	n/a	https://biogas.ifas.ufl.edu/Internships/2011/files/kalvin.pdf<br>https://www.gminsights.com/industry-analysis/glycerol-market-size
Peptides (e.g. Bioactive)	Nutrition & Dietary Supplements	Applied Research	117.3 B (2024)	10.77% (2025-2030)	n/a	n/a	https://www.mdpi.com/2673-6209/4/3/35<br>https://www.grandviewresearch.com/industry-analysis/peptide-therapeutics-market
Protein-enriched food ingredients	Nutrition & Dietary Supplements	Applied Research	52.6 B (2024)	5.6% (2025-2033)	n/a	n/a	https://www.frontiersin.org/articles/10.3389/fnut.2021.737816/full<br>https://www.grandviewresearch.com/industry-analysis/protein-ingredients-market
Algal polysaccharides	Nutrition & Dietary Supplements	Commercial	15.2 B (2023)	5.0% (2025-2032)	1.2 B (2023)	9.0% (2023-2033)	https://www.mdpi.com/2673-4176/2/4/46<br>https://www.skyquestt.com/report/polysaccharides-market<br>https://www.factmr.com/report/marine-algae-polysaccharides-market
Antioxidant additives	Nutrition & Dietary Supplements	Commercial	4.1 B (2024)	6.86% (2025-2043)	n/a	n/a	https://www.fortunebusinessinsights.com/industry-reports/food-antioxidants-market-100789
Dietary supplements	Nutrition & Dietary Supplements	Commercial	192.6 B (2024)	8.9% (2025-2033)	2.52 B (2025)	7.3% (2025-2035)	Dietary Supplements Market Size | Industry Report, 2033<br>https://www.futuremarketinsights.com/reports/algae-based-supplement-market
