# Supplementary figures and images for: Analytical Framework to Navigate Microalgae-Based Product Development—Aligning Commercialization and Regulatory Pathways
Source: Mar Drugs. 2026 Feb 3;24(2):66. doi: 10.3390/md24020066 (PMC12941414; doi:10.3390/md24020066)

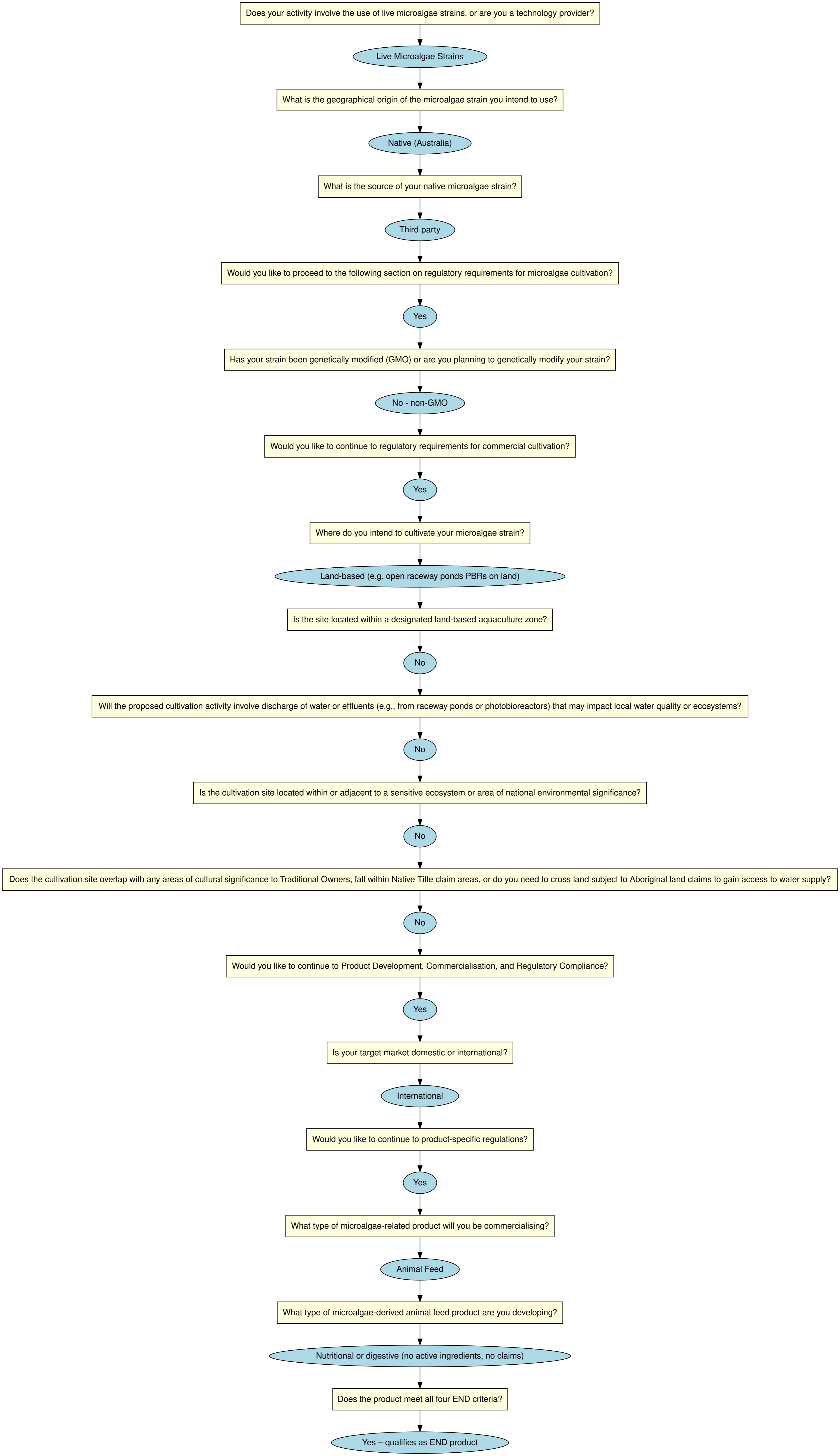

Supplement: Supplementary file 1 [file marinedrugs-24-00066-s001.zip › Figure S1-Regulatory pathway for commercial production and export of microalgae END feed.pdf]

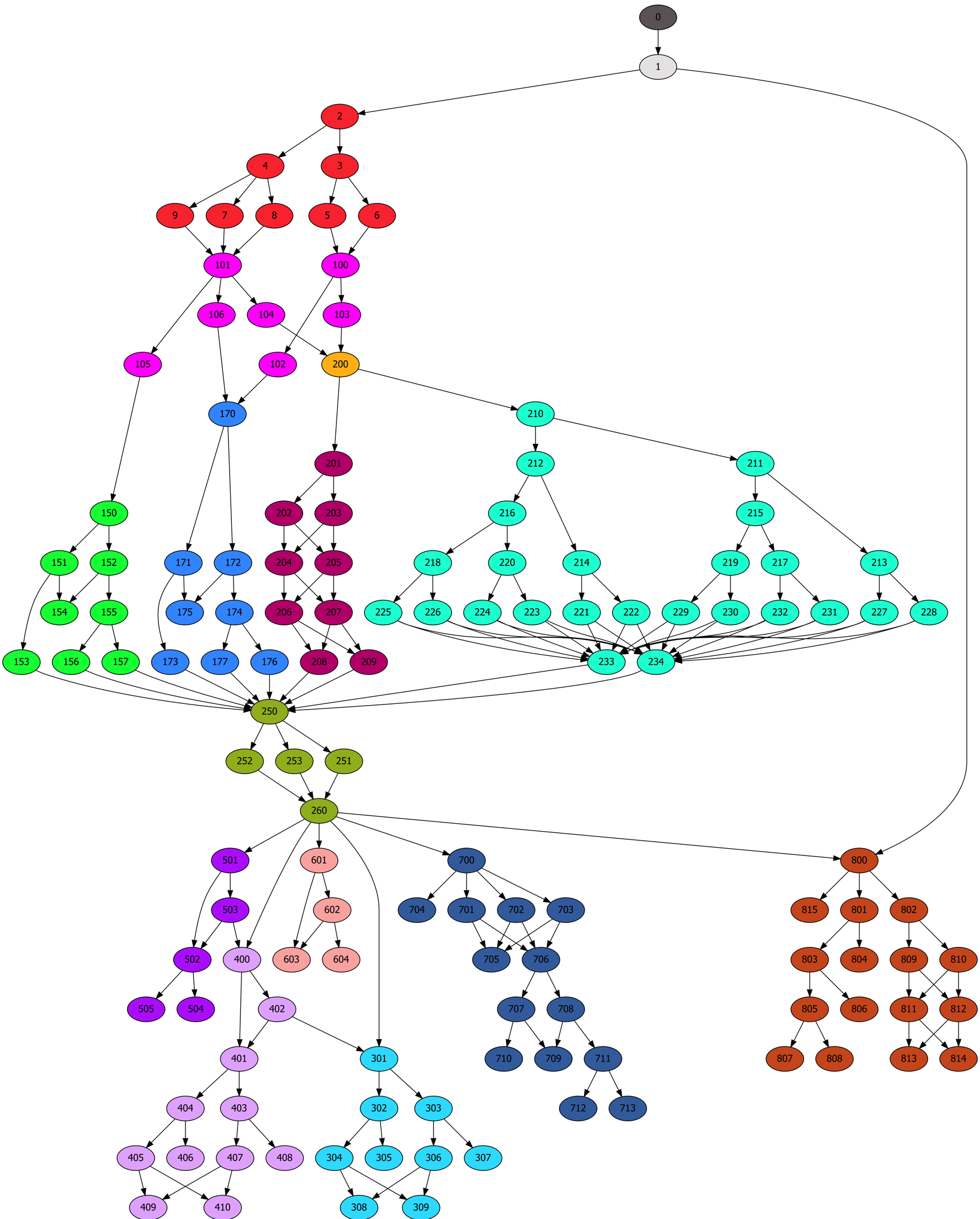

Supplement: Supplementary file 1 [file marinedrugs-24-00066-s001.zip › Figure S2-Full regulatory pathway nodes for microalgae in Australia.pdf]
